# “I haven’t had to bare my soul but now I kind of have to”: describing how voluntary assisted dying conscientious objectors anticipated approaching conversations with patients in Victoria, Australia

**DOI:** 10.1186/s12910-021-00717-0

**Published:** 2021-11-12

**Authors:** Casey Michelle Haining, Louise Anne Keogh

**Affiliations:** Centre for Health Equity, Melbourne School of Population and Global Health, University of Melbourne, Victoria, 3010 Australia

**Keywords:** Voluntary assisted dying, Conscientious objection, Bioethics, Health professionals, Qualitative research

## Abstract

**Background:**

Dealing with end of life is challenging for patients and health professionals alike. The situation becomes even more challenging when a patient requests a legally permitted medical service that a health professional is unable to provide due to a conflict of conscience. Such a scenario arises when Victorian health professionals, with a conscientious objection (CO) to voluntary assisted dying (VAD), are presented with patients who request VAD or merely ask about VAD. The *Voluntary Assisted Dying Act 2017* (Vic) recognizes the inherent conflict of conscience that may arise for some health professionals when asked to provide VAD and responds by affording broad protection to conscientious objectors who wish to refuse to take part in the VAD process.

**Methods:**

Seventeen semi-structured qualitative interviews were conducted with Victorian health professionals with a self-identified CO to VAD in the lead-up to the implementation of VAD in Victoria. Interviews explored how participants anticipated they would manage their CO in practice. Interviews were transcribed verbatim and analyzed thematically.

**Results:**

Our results reveal that the way in which health professionals claimed they would approach CO conversations is variable and was dependant on the strength of their opposition to VAD. We categorized conscientious objectors according to their approach as either dissuasive non-referrers, passive non-referrers, facilitators or negotiators. Our study also explores the perceived difficulties of exercising one’s CO as identified by our participants.

**Conclusion:**

The broad protection offered by the *Voluntary Assisted Dying Act 2017* (Vic) encourages a range of behaviors from conscientious objectors, due to the minimal obligations imposed. In order to assist conscientious objectors, more policy, institutional guidance, and education needs to be available to conscientious objectors explicitly addressing how to effectively manage one’s CO. Such guidance is imperative to ensuring that their moral integrity is preserved and that they are exercising their CO appropriately.

## Background

Navigating end-of-life medical care is challenging for health professionals and patients alike. Patients with terminal illnesses are often grappling with intractable symptoms, balancing hope and realism, and are yearning for some control over their symptoms [1]. The therapeutic relationship thrives on mutual respect and trust between the health professional and the patient [2]. However, tension is likely to arise when a patient presents to a health professional, often in hopeless circumstances, and the health professional is unable to directly give the patient the treatment they request, due to a conflict in conscience [2]. Such a quandary may surface when health professionals, who hold a conscientious objection (CO) to voluntary assisted dying (VAD), are presented with patients who request VAD or merely ask about VAD.

CO in health care occurs when health professionals exempt themselves from providing or participating in a legally permitted health service on moral, religious or philosophical grounds [3]. Traditionally CO claims were thought to be the product of religious convictions; however, there is evidence to suggest that the motivations behind one’s CO are diverse and may stem from both religious and secular convictions [4]. It is postulated that by permitting health professionals to claim a CO, they will be able to deliver health care without compromising their moral integrity [5]. Moral integrity is thought to have intrinsic value to a person and is imperative for  maintaining one’s self-worth and identity [5]. A loss of moral integrity can have devasting effects and can result in a health professional experiencing strong feelings of remorse, guilt and shame [5], which not only has dire effects for the health professional, but could, in theory, lead to the delivery of suboptimal health care.

Whether or not  CO should be permitted in the healthcare setting is a point of contention and is debated at length in the literature. Some commentators support the view of conscience absolutism [6, 7]. According to this view, health professionals should not be required to provide a good or service that violates their conscience and therefore should not be obligated to directly or indirectly participate in its provision nor expected to facilitate access to it [8]. Conversely, some commentators posit that CO has no place in medicine [9–13]. Subscribers of this position often argue that when a legal medical service is requested by patients, health professionals should provide it, especially when they have a monopoly on provision [11]. Within these two extreme positions, is what is known as the ‘compromise position.’ Proponents of this position argue that CO should be permitted provided additional obligations are placed on conscientious objectors, such as an obligation to provide an effective referral, to justify one’s CO in terms of reasonability or genuineness, and/or declare their CO to the patient [14–18].

### The legal context of VAD in Victoria

**Fig. 1:**
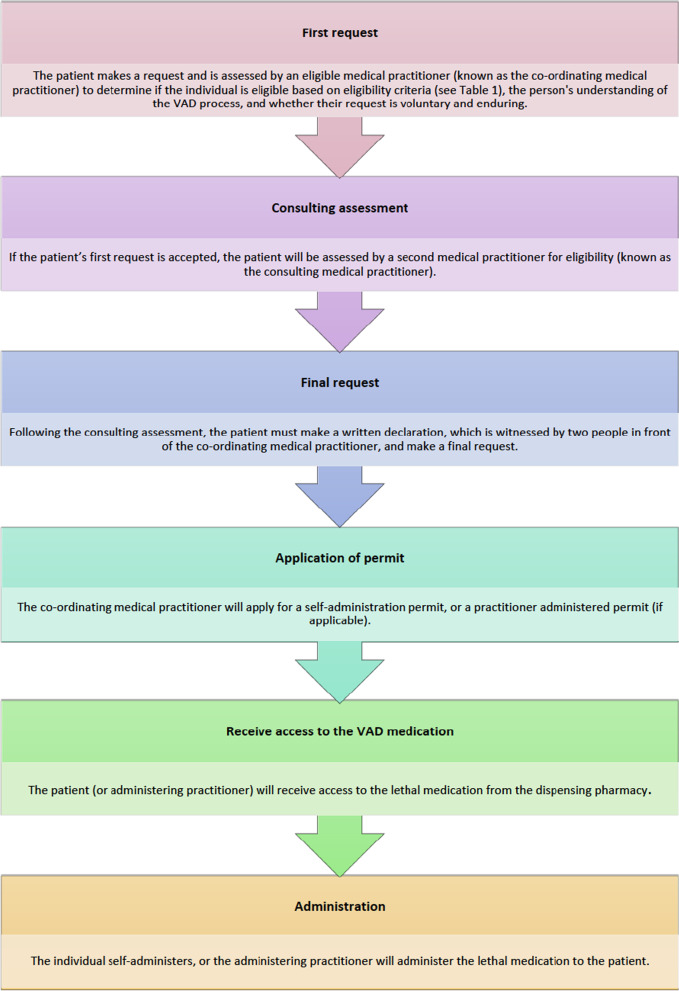
Summary of the VAD process (image created by the authors)

In Australia, at the time of writing, VAD legislation has passed in four states namely Victoria, Western Australia, Tasmania and South Australia; however, VAD legislation has only commenced in Victoria and Western Australia to date [19–22]. Of concern in this article, is the Victorian *Voluntary Assisted Dying Act* 2017 (hereafter the ‘Act’) [19]. VAD in Victoria largely relies on a self-administration model (see Fig. 1 for a brief summary of the VAD process) and is only accessible to patients, who have decision-making capacity in relation to VAD, and who satisfy the strict eligibility criteria prescribed by section nine of the Act (see Table 1). In the event that the patient is physically incapable of self-administration or digestion of the VAD substance, a medical practitioner can apply for a practitioner administration permit allowing them to administer the substance to the patient.

**Table 1:** Eligibility criteria

Age	Must be over eighteen
Residency requirement	The individual must have resided in Victoria for at least twelve months, and is either an Australian citizen or permanent resident
Disease characteristics	Incurable, advanced and progressive Is expected to cause death within six months, or twelve months in the case of neurodegenerative diseases Must cause the individual suffering, which is unable to be relieved in a manner tolerable to the person

In the context of VAD, Australian states accommodate conscientious objectors by affording them legal protection through the inclusion of CO provisions (sometimes referred to as conscience clauses) in domestic legislation. Whilst all the Australian jurisdictions, who have passed VAD laws, formally enshrine the right to CO in the form of a CO provision in their legislation (current or future), the framing and scope  of these provisions varies across jurisdictions. The CO provision in the Victorian Act is section seven. Section seven offers broad protection to registered medical practitioners by exempting them from providing information about VAD; participating in the request and assessment process; applying for a VAD permit; supplying, prescribing or administering the VAD substance; being present at the time of VAD administration; and dispensing a prescription for the VAD substance [19]. In fact, the Act only requires that the medical practitioner inform the patient of their refusal and, in the event that the consulting practitioner refuses the referral for a consulting assessment, the co-ordinating practitioner as well, within seven days.

Section seven’s framing contrasts with the positions adopted in other Australian jurisdictions. Although similar obligations apply with respect to informing the patient and the co-ordinating practitioner (or equivalent) in all jurisdictions, there are some notable differences. For instance, in Western Australia the notification of refusal must be immediate, and if the patient is refused on the first request, the practitioner must supply the patient with prescribed information about VAD [20]. The Tasmanian Act requires the conscientious objector to provide the patient with the contact details of the VAD Commission, regardless of their CO [21]. In Tasmania, the Commission is intended to serve a number of functions, among them is “to provide an appropriate level of assistance to persons who wish to access voluntary assisted dying but who are prevented from, or hampered in, accessing the process” [21]. The South Australian Act extends its protections to institutional objections, namely health service establishments. However, under the South Australian Act, the relevant health service provider who operates the establishment must, upon advising the patient of the institution’s refusal, transfer the patient to another health service provider and take reasonable steps to facilitate the transfer [22]. The same institutional protections, however, do not extend to residential premises such as nursing homes, residential aged care facilities and retirement villages [22]. Generally, such premises must allow residents to access information about VAD and make VAD requests, and have additional obligations to permit and/or facilitate this [22].

Although not prominent in VAD laws in Australia, referral obligations that apply to individual medical practitioners claiming a CO do exist in Australian abortion law. Some Australian jurisdictions mandate that conscientious objectors to abortion provide a direct referral to an individual practitioner or health provider who does not have a CO [23–25] or provide an indirect referral where patients are given a list of prescribed services [26], with some jurisdictions permitting a combination of approaches [27, 28].

### CO in practice

Whilst it is important to understand the legal framework that governs CO, it is also important to understand how CO operates in practice. During the 18-month implementation period before VAD became operational, CO was identified as one of the implementation challenges [29]. While there is literature that examine CO and assisted dying [4, 30–32], there are limited empirical studies that examine how exactly health professionals with CO to VAD approach conversations with patients requesting or merely asking about VAD. The majority of studies that examine how health professionals exercise their CO are conducted in the context of abortion and accordingly have limited generalizability to VAD.

During the implementation of VAD, it was envisaged that some health professionals will make a global decision about providing VAD, where others would adopt a case-by case approach [29]. This prediction is consistent with the findings of empirical studies conducted in the abortion context which find that the manner in which individual health professionals will exercise their CO differs [33–36]. Some commentators have suggested that the range of different behaviors health professionals exhibit when exercising their CO is best conceptualized as operating across a continuum [33, 36]. Fink and colleagues (2016) created a typology of conscientious objectors based on qualitative interviews with Colombian physicians, who had a self-identified CO to abortion, identifying three types of objectors: extreme, moderate and partial. At one end of the spectrum, *‘extreme objectors’* were described as individuals who refused to perform abortions under any circumstances, refused to provide referrals and actively dissuaded their patients from accessing abortion services through the provision of inaccurate medical and legal information [33]. On the other end of the spectrum, *‘partial objectors’* were found to adopt a case-by-case approach and would refer their patients [33].

Furthermore, there is evidence that CO, in some cases, is poorly regulated and often utilized in an ad hoc manner [35, 37, 38]. This is thought to be, at least in part, due to the limited guidance and absence of formal guidelines offered to health professionals [35]. Some health professionals are even unaware of the term CO [38]. Evidence also suggests that health professionals are not satisfying their legal obligations with respect to CO, which may be the product of a deliberative breach or ignorance [35, 39]. The consequence of inappropriate use of CO is that it hampers patients’ ability to access a legally permitted service [35, 39].

During the implementation of VAD it was clear some health professionals would be claiming a CO, with suggestion that the option to conscientiously object was fundamental to mitigating pressure on health professionals [40]. However, commentators expressed concern about how CO would operate and be managed in practice. For instance, some expressed concern about how the patient-doctor relationship would be maintained if a health professional elected not to participate [41]. One study found that there were concerns that there would be pressure to participate, despite the fact health professionals were legally permitted to opt out [42].

Section seven, outside of affording broad protection, failed to establish a framework for respecting conscience [29] and offered limited guidance in relation to how health practitioners should appropriately claim a CO, apart from requiring practitioners to inform the patient that they refuse their request (and co-ordinating practitioner, if appropriate). Victoria’s Department of Health and Human Services has subsequently provided some guidance around CO [43]; however, it is acknowledged that such guidance represents best practice rather than explicit legal obligations. The guidance encourages health practitioners to tell their employer/supervisor of their CO to assist the health service in understanding the views of its staff and helping to manage access [43]. The guidelines also stipulate that despite one’s CO, health practitioners should demonstrate a willingness to listen, empathize with and support patients to make informed decisions; respect their patient’s autonomy and choice regardless of conflicts of conscience; and provide routine and other care unrelated to the VAD request [43]. The guidelines also reinforce that health practitioners should not impede access to treatment and encourage health practitioners to refer their patients to another health practitioner; however, the guidance identifies that not all medical practitioners will be comfortable doing so [43].

Furthermore, the Victorian Government recognized that CO may compromise access to VAD and has established a Statewide Voluntary Assisted Dying Care Navigation Service to mitigate this risk. The service helps to facilitate patient access to VAD by putting requesting patients in contact with medical practitioners, who have completed the VAD training [44]. However, the effectiveness of this service is likely to be contingent upon the availability of willing providers in the patient’s geographical proximity and accordingly the high rates of CO that tend to exist in regional and rural Victoria [37] may limit the effectiveness of this service.

Whilst some legislative and policy guidance does exist around CO, little is known about how CO is managed by health practitioners with a CO in the VAD context; specifically how conscientious objectors exercise their CO in a way that preserves their moral integrity to the greatest extent possible, whilst still enabling them to fulfill their professional obligations to not impede access to a medical procedure they object to. The authors sought to bridge the current gap in the literature by investigating how health professionals envisaged they would manage their CO to VAD in practice, in the lead-up to the legalization of VAD in Victoria, in June 2019.

## Methods

The study design featured a qualitative, phenomenological methodology that permitted the authors to get an insight into the lived experiences of the participants [45], through the use of semi-structured interviews. To be eligible to participate, individuals needed to be practising in the health profession, in Victoria, when VAD was set to be implemented (i.e. June 2019) and had to identify as having a CO to VAD. We advertised our study via religious and secular health organizations and specialists’ colleges, who were willing to advertise our study via their communication channels free of charge. We also utilized the authors’ professional networks to assist with advertising. Interested participants were asked to contact CMH via phone or email and were subsequently provided with a plain language statement to provide further information about the study and a consent form. We used snowball sampling to extend our sample size.

### Data collection

Seventeen semi-structured interviews were carried out by CMH either in person or on the phone in the lead-up to VAD implementation. The interviews were audio-recorded and ranged between thirty-eight and ninety minutes. Each transcript was de-identified and transcribed verbatim by CMH. Participants were actively recruited between June and August 2018, with the last interview carried out in February 2019.

### Data analysis

Thematic analysis was utilized in order to identify patterns and themes in the data, and fully explain any variation present [46]. CMH and LAK initially read through each transcript and developed a coding framework. The framework was tested on a subset of transcripts and was subsequently refined to develop the final coding framework. The framework consisted of four top-level codes including: motivation for CO; management of CO; perceptions of the VAD legislation; and future implementation of VAD. In order to test for saturation, the code ‘motivation for CO’ was used. Data analysis of this code has been published elsewhere [4]. Once no new sub-themes emerged from the transcripts, saturation was deemed to be reached. *N Vivo 12* was used in order to code each of the transcripts. Each code and sub-code were then fully analyzed in order to summarize the data. This paper will explore the code ‘management of CO,’ specifically the sub-code ‘CO conversations with patients.’

### Research ethics

The study was approved by the University of Melbourne’s Human Ethics Advisory Group (1851586.1). Each participant provided informed consent to participate in this study either by written or verbal consent. Both forms of consent were approved by the ethics committee. Most participants provided written consent. Some participants, whose interviews were conducted over the phone, elected to provide verbal consent. Participants who wanted to give verbal consent were read out each statement in the written consent form and asked whether or not they consented to participating. All aspects of this study were carried out in accordance with our research protocol (approved by the ethics committee) and the National Statement on Ethical Conduct in Human Research (Australia) [47].

## Results

First, we will examine the commonalities in how the health professionals anticipated they would approach conversations with patients and how they would declare their CO to patients. Secondly, we will explore how the perceived conduct of the self-identified conscientious objectors can be categorized based on commonalities between participants’ approaches. Finally, the results will examine some of perceived difficulties that participants raised when asked to consider how they envisaged managing their CO.

### Sample

Seventeen health professionals participated in the study (Table 2). Each participant was assigned a pseudonym.

**Table 2:** Participant characteristics (n = 17)

Characteristic	Number
Gender	
Female	9
Male	8
Health professional role	
Allied health professional	1
Doctor	15
Nurse	1
Speciality	
General practice	4
Geriatrics	1
Intensive care	1
Oncology	5
Palliative care	5
Pathology	1
Number of years in the profession	
Less than 15 years	10
Greater than 15 years	7
Geographical location	
Metropolitan	14
Regional	3
CO approach (see Fig. 2 below)	
Dissuasive non-referrer	2
Passive non-referrer	6
Facilitator	7
Negotiator	2

### Commonality: unpacking symptoms

A commonality found across all groups was that each health professional was willing to unpack the patient’s symptoms and explore their desire to die. Drawing on their lived experiences, many participants revealed that a patient’s desire to end their life was not uncommon and was occurring before VAD was legally permitted. They felt that such desires, however, were often flippant and transient in nature.

Participants felt the best way of addressing a patient’s wish to access VAD was to first unpack their symptoms. It was thought that by unpacking a patient’s symptoms there may be some underlying problems outside the individual’s illness that can be addressed, and once such problems are resolved the patient’s desire to die would subside.

I suppose really, we say it is a cry for help. What is the problem in that person’s life that is causing them to feel like that? So, is it pain? Is it stress at home? Is it an argument with a son? Is it they’re lonely? Is it that they can’t care for their house? Is it their dog has died? Is it an incurable illness? … What’s driving it? … Why are they thinking that? Are they depressed? … [When] things are explored and gone through most [people] revoke their requests. **[P4]**

Despite a health professional’s reservations towards VAD, it was considered to be imperative that the patient’s symptoms were explored; failing to do so would inadvertently shut the patient down.

[To] have clinicians say up front, well look I don’t want to do that sort of thing … completely shuts down the conversation … it might not be a sustained wish to die. It could be a lot of other things. It should be explored. **[P11]**

It was acknowledged, however, that there was currently limited scope for medical interventions to attend to a patient’s psychosocial distress.

With the physical symptoms there is a lot we can do. In terms of psychosocial and psychological distress, I think some of our interventions might be limited and psychosocial interventions are incredibly limited in terms of meaning of life and burden on caregivers and things like that … I’m not sure they have a medical solution and I guess that is where a bit of a discrepancy exists as to what I can physically do to change people’s experience of the dying process. **[P8]**

### Declaring one’s CO

When participants were asked how they would inform their patients about their CO, a range of different approaches were identified. However, all participants indicated that they would communicate their objection to the patient at some point in the conversation. For some participants a short, simple, and direct approach was deemed most appropriate. Others preferred to use another reason for their objection to involvement. Most participants indicated that they would not go into detail about what motivates their CO to patients.

I think simple is best … because the patient doesn’t need to know anything I just told you ... they just need to know that I have an objection to be involved in this.** [P3]**

I will have to develop myself a little conversation to gently explain, or if asked why… I will effectively have to keep it short and say “look, I can’t be involved and someone else would have to.” **[P6]**

Some participants indicated that before declaring their CO, in addition to unpacking the patient’s symptoms, it is important that they try and understand the patient’s story.

I think it is simple enough to explain yourself … in a non-judgemental way … I don’t declare from the outset my discomfort … I will wait to listen for a situation or story and then I will … if it’s getting to a point where I’m uncomfortable, then I have to say, “look, fair enough, this is the way you wish to proceed, but this is where I stand, this is a personal thing.”**[P2]**

Some participants explained that it is imperative that the patient understood that the health professional is not condemning the patient’s decision. Participants claimed that they would assure the patient that they did not have a problem with their desire to access VAD, but would make it clear to them that they were not willing to be a practitioner directly involved.

I will be very open with them … I don’t have a problem with them wanting to access it and make a promise to care for them before and their families after, but … I’m not going to be one of the two doctors arranging it.**[P5]**

Other participants suggested they would adopt an approach analogous to that of refusing futile treatment. As such, they would declare their CO by suggesting that VAD was of no utility to them.

I would say “look, I cannot see any reason you should do this. I just don’t want to be part of it … I’m sorry, I can’t help you."**[P10]**

One participant noted that they would use the fact that they did not undergo the requisite training to notify their patients that they will not participate in the VAD process.

I will never do the training that will make me eligible to assist … so, therefore, I would just say to the patient “I do not have the adequate training. You’ll have to see somebody else.” **[P17]**

In a similar vein, it was raised that being employed by a hospital, that has publicly declared that they will not provide VAD, would assist a health professional to declare their CO to the patient.

I currently work in a hospital … that has developed a position statement as an organization that they will not provide the service within their institution … I think that is fairly publicly known and so I would give recourse to that. **[P16]**

### Four different approaches to communication with patients seeking VAD

**Fig. 2:**
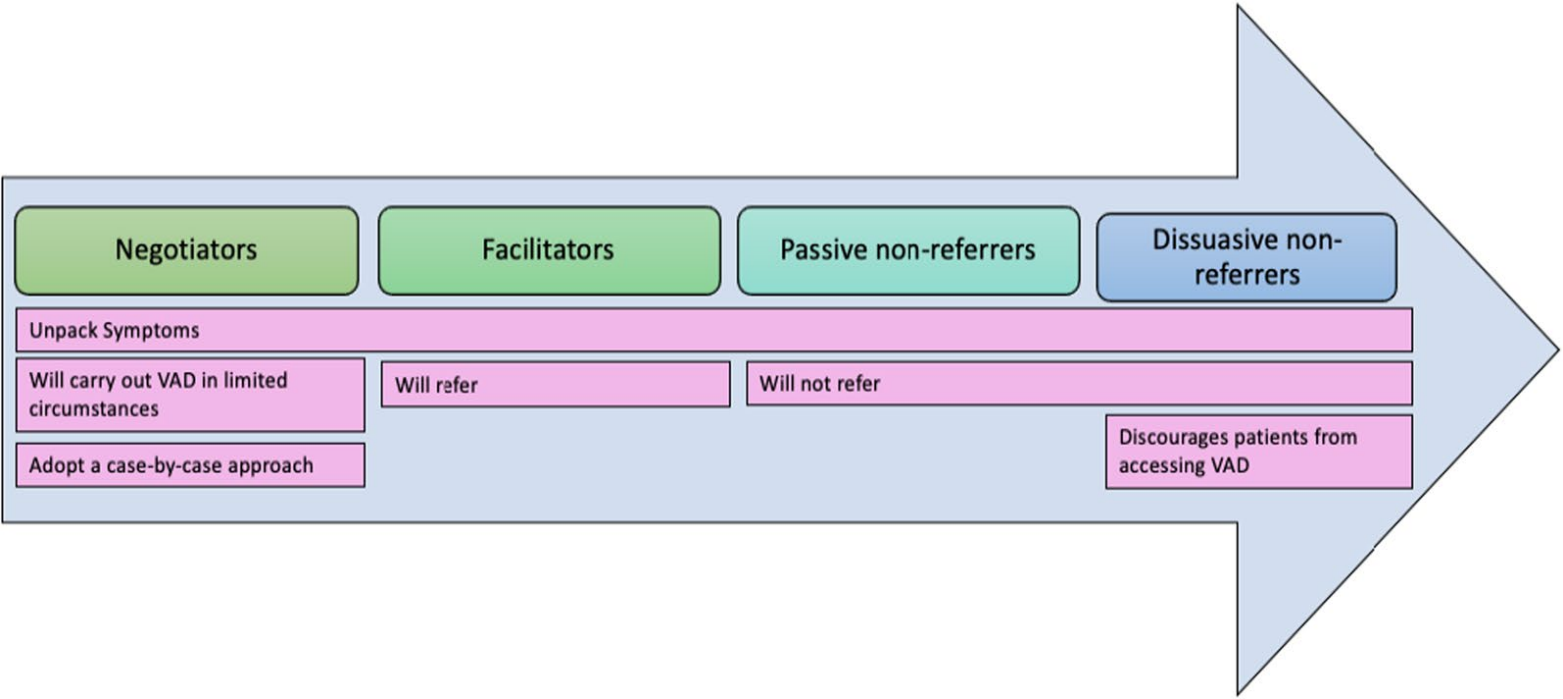
Change in behavior of conscientious objectors as strength of opposition to patient access to VAD increases (image created by authors)

Data analysis revealed that there were broadly four overarching profiles found in this study: dissuasive non-referrers, passive non-referrers, facilitators and negotiators. A more accurate depiction would represent conscientious objectors as occupying a series of dynamic positions on a continuum. Figure 2 depicts the four different approaches. Where participants are positioned on the continuum is thought to be dependent on the strength of their CO and degree in which they are willing to be complicit in the VAD process.

### Dissuasive non-referrers (n = 2)

Participants categorized as dissuasive non-referrers disclosed that they would try and discourage patients from pursuing the VAD route. They would encourage patients to pursue alternatives to VAD such as palliative care or try and give patients extra time to reconsider their decision. The dissuasive non-referrers would also provide the patients with reasons why they should not pursue the VAD route. Despite claiming they would actively dissuade their patients, there was no indication that they would condemn patients or provide misleading/inaccurate information.

I would do everything in my power… to talk them out of it, until the point where … genuinely … the person, despite my best efforts and information, really wanted it … I don’t believe in adding to the person’s burden. If … despite everything that I said or thought, the person is just one of those people that really wants it … I would say “well, I’ve done the best I can” and let them go. But I would go out of my way to try and convince them. I would give them extra time. I would try and provide them literature or reasons. I would ask them to consider … take time for their relatives to come in and see me. **[P4]**

Despite the apparent dissonance between beneficence (as defined by the health professional and what they believed to be in the patient’s best interests) and patient autonomy, these participants emphasized that they ultimately respected patient autonomy and would only do what they considered to be reasonable to deter their patients from VAD. If participants were adamant that they still wanted to hasten their death, despite the dissuasive non-referrer’s best attempt to dissuade the patient, they would respect their patients wishes, but would not actively facilitate a referral.

I’m not into harassing the person, I would take it as far as I responsibly could and then have to desist and accept that the person [is] perhaps one of the rare breeds that really want it … I do actually respect people’s autonomy and I do actually believe that [for] a very small number of people, it could be the right decision. **[P4]**

### Passive non-referrers (n = 6)

Non-referrers indicated that they would not facilitate a referral for their patients to a colleague, who would willingly provide VAD access. This group differed from the dissuasive non-referrers in the sense that they would not actively dissuade patients. Despite not being personally responsible for signing off on a patient’s VAD access, these participants felt that by actively providing a referral they would be complicit in the process and have their moral integrity compromised.

I’m comfortable with there not being a referral requirement … because I think that the process … has significant implications for both the patient and a participating doctor, and I don’t feel that it is appropriate to mandate that the medical professional intentionally cause death to their patients. **[P16]**

Some participants would say to their patients “you can see someone else.” However, for the purposes of the authors’ categorization this was not considered a formal referral, as no active steps were taken to facilitate or formalize the referral.

Using the analogy of termination of pregnancy, one health professional stated:

It still implicates them though, because … it still forces them to make a referral, a formal referral … I’m not comfortable organizing a formal referral letter that allows a patient to see a doctor, who I know is going to terminate their pregnancy. Well, you may say you’re picking a little bit, what is the difference between you writing a formal letter and you saying to them you have to see my colleague? Well, look for me, there is a difference. I’m not swaying my colleague either way and I’m not formally becoming involved in the process, but I have to respect their rights at the end of day. I can’t dismiss it if a patient seeks that … they are [in] every way entitled to their right, but that for me is how I balance it. **[P2]**

### Facilitators (n = 7)

Conscientious objectors categorized as facilitators were those health professionals, who despite not wanting to provide VAD themselves, were still willing to assist patients in finding a willing provider.

I probably would refer to someone, if it was possible to know who … would be happy to participate in [the VAD] process. **[P8]**

Despite not being obligated to, the facilitators were committed to ensuring that patients had access to a legally permitted medical service through referral. These health professionals highlighted that they did not want to “block” patients.

The VAD laws …[don’t] actually mandate that you have to refer on, but I will, because it’s what my patient wants … that’s part of me caring for them as a whole … I will be trying to find colleagues, who I trust, and who are going to be able to do that system, to negotiate that VAD system fairly … and provide them with information they need. **[P5]**

### Negotiators (n = 2)

The negotiators represented a category of conscientious objectors who indicated that they would adopt a case-by-case approach and would facilitate VAD access in limited circumstances. However, their decision to permit VAD access would not be considered lightly. The negotiators would be cautious with their approach, but were hesitant about adopting a blanket approach, whereby they either never offered VAD or offered VAD every time a patient was eligible.

I think I have five people so far … I don’t think willingly is the right word, but I think I would solemnly perform euthanasia for them and that’s how I view abortion as well. There are certain situations that I would be solemnly obliged to perform an abortion ... and so I can use it by the same yardstick … I think it is case-by-case and it really should be case-by-case … I’m very worried about a blanket approach. **[P13]**

When asked about what factors were important to the health professional when considering whether or not they would facilitate VAD, one participant indicated:

The most important thing for me is time …. We need time to establish that euthanasia is the right way … [We] need to have attempts made by this person to improve their life, and then sufficient amount time to take place until you then decide that euthanasia is the diagnosis for them ... I don’t think you can make it too long … people will give out. I would like to see longer, but I think you have to be realistic because it might be what people actually want, so I would give them months from the time that they meet a specialist. **[P13]**

### Perceived difficulties for all types of conscientious objector approaches

Regardless of the conscientious objector approach, most participants acknowledged how challenging it would be to tell their patient that they were not willing to provide them with a particular treatment. These conversations were thought to be uncomfortable for both the health professional and end-of-life patient, who is likely to be vulnerable.

Don’t underestimate how difficult these conversations are. They tax you to n^th^ degree ... No, it won’t be comfortable, but it needs to be done.** [P11]**

I will do what I always do and spend time with them, but there will always be a part of me that will feel internally ill, because I don’t think this is the best thing for our society. Having said that, I’m going to have to sit with it … [and] at the moment it is creating anxiety. **[P7]**

Some participants identified that their discomfort arose as a result of a feeling that they were letting their patients down.

If a patient asks me to do cartwheels down the hallway, I would probably do it because that is how we’re built. You don’t get into this job, if you aren’t built like that. So that is the problem for me, the real tension between wanting to help in whatever way the patient would feel to be most help to them, but just intrinsically being unable to do what I have been asked. **[P9]**

I don’t like the idea of having to turn a patient away and say, “I can’t help you.” ... I also can’t continue to help them in that situation. In all honesty, I have no idea about how I am going to manage it. **[P6]**

As previously noted, requests for a hastened death were not uncommon amongst patients with terminal illnesses prior to the legalization of VAD. However, since assisted dying was previously illegal, health professionals were able to rely on the legal barrier to deny requests. However, given VAD was going to be lawful, health professionals could no longer rely on the legal barrier to deny a request. This proved to be disconcerting for participants.

It has been easy because I have been able to say it is illegal, and that has been very straight forward, and I haven’t had to bare my soul but now I kind of have to. **[P9]**

## Discussion

This study sought to examine how health professionals anticipated they would manage their CO to VAD in practice. Many participants described how difficult exercising their CO would be. Indeed, for many of participants refusing to provide a medical service was unprecedented. The broad framing of section seven in effect accommodates a diverse range of health professional approaches to CO conversations. Section seven essentially conforms to what has been described as the conscience absolutism view of CO [5]. This framing aims to protect healthcare practitioners from performing a medical service that would conflict with their conscience and offers further protection by not requiring them to provide information or refer the patient to a willing colleague [5].

Consistent with Fink and colleagues (2016) [33], our participants could be categorized into different ‘conscientious objector types’ based upon how participants envisaged they would exercise their CO in practice. Such a typology, however, is necessarily reductionist and relies on a two-dimensional representation of complex phenomenon. The categorization inevitably frames the different CO profiles as being static due to the specific characteristics that the authors have selected. A more accurate depiction, as has been found in other studies, would be to represent conscientious objectors as occupying a series of dynamic positions on a continuum [33, 36]. It is postulated that where participants fit on the continuum is contingent upon the participant’s interpretation of the moral acceptability of participating in the VAD process and the degree in which they were willing to be complicit in the process, which would ultimately be informed by the strength of the individual’s CO.

Notably, none of our participants resembled Fink’s ‘extreme conscientious objector’ [33]. Whilst two participants suggested they may attempt to dissuade patients, they reasoned they would do so because VAD was thought to be a dangerous or inappropriate process for the vast majority of patients. However, they did not indicate that they would give the patient inaccurate or false medical and legal information like the ‘extreme conscientious objectors’ [33]. The dissuasive non-referrers, despite discouraging VAD, did not see themselves as imposing their beliefs on patients. Their approach highlighted a tension between beneficence and patient autonomy. According to these participants, the patient’s best interests would not be served by VAD and hence would actively discourage patients from accessing VAD. However, participants anticipated that in cases where their patients were adamant VAD was the right avenue for them to pursue, even despite the participant’s best efforts to discourage them, the participant would ultimately prioritize and respect the patient’s autonomy and “let them go.”

There was a general consensus amongst the dissuasive non-referrers and passive non-referrers that VAD was not acceptable and accordingly it would be ‘wrong’ for them to take any part in the process. Even if they were not going to be facilitating VAD access, referral was still viewed as being complicit in the wrongdoing. Accordingly, not referring patients was seen as a mechanism that assists in protecting a health professional’s conscience and moral integrity [48].

The majority of our participants could be construed as facilitators. Whilst the current framing of section seven does not impose a positive obligation on participants to refer their patients onto a willing practitioner, these participants felt that providing a referral was their responsibility, given they could not, due to conflicts in conscience, offer VAD personally. For these participants, referring seemed to be a principled way in which they were able to enable access to VAD, without directly participating in a service they fundamentally disagreed with [49]. For a subset of these participants, there was some suggestion that they were accepting of the fact that VAD was introduced through a representative democratic process, and hence were happy to facilitate a patient’s access to a legally permitted service through referral, but were not prepared to directly implicate themselves.

Despite their reservations and concerns about the VAD process, negotiators seemed to feel somewhat obligated to facilitate a patient's request in a subset of circumstances. For these participants, each patient should in essence be assessed for VAD access on the ‘merits’ of their case. Ultimately the health professional’s decision to grant VAD access would be based upon the judgement of the particular health professional and whether or not that health professional would be willing to ‘sign off’ on the patient. The negotiators were hesitant to take a firm position on VAD. They were not prepared to always facilitate a patient’s access if they satisfied the prescribed eligibility criteria. Rather these health professionals seemed to have devised their own criteria, to determine whether VAD would be appropriate or morally acceptable for a particular patient. This approach inevitably introduces a degree of subjectivity and does introduce a degree of paternalism. It has been previously argued that a case-by-case approach is an improper use of CO, because the denial of the health service results from the health professional not agreeing with the patient’s decision, rather than stemming from conscience grounds [50]. Our participants provided a slightly nuanced perspective on this, suggesting that there would be certain clinical presentations where they felt VAD may be in the patient’s best interests, and where they felt “solemnly obliged” to partake in the VAD process. For these participants, whether or not their conscience was perturbed would be informed by the patient’s specific circumstances, which would ultimately dictate whether they felt they could conscionably partake in the VAD process.

### Study limitations

Despite the authors’ attempts to recruit as many health professionals as possible, the sample in this study is ultimately small and hence the results should be interpreted in the context of such a limitation. Similarly, whilst the authors tried to minimize bias in sampling by recruiting from a number of health organizations and colleges, we were only able to advertise via organizations and colleges who were willing to advertise our study free of charge, given no funding was attached to this research. As a result, some specialities may have been underrepresented. Specialities such as psychiatry did not appear to be captured by our recruitment strategy and warrants targeted recruitment in the future. Moreover, as a result of snowball sampling, some specialities were overrepresented.

Furthermore, our study did not find a ‘extreme conscientious objector,’ as defined by Fink et al. (2016) [33]; however, we are unable to conclude with confidence that such individuals that fit this profile do not exist in Victoria. It is likely that such conscientious objectors do exist, but due to the sensitivity of the topic under examination may have been discouraged from participating in our study.

Moreover, this study was conducted prior to the implementation of VAD in Victoria, and hence how CO was envisaged to operate in practice may differ to how it is has been carried out post-implementation. A comparative study comparing pre- and post-implementation of VAD and CO management is an area that warrants future investigation.

## Conclusion

How CO to VAD is managed in practice has been understudied empirically. This study suggests conscientious objectors can be categorized based on four different approaches to CO conversations as either dissuasive non-referrers, passive non-referrers, facilitators or negotiators. Despite the defining characteristics existing amongst the conscientious objectors, their behaviors are best conceptualized as operating across a continuum. The broad framing of the Victorian VAD CO provision somewhat encourages such a diversity in behaviors, due to the minimal obligations on health professionals when carrying out their CO. It is not within this article’s ambit to evaluate the appropriateness of CO behaviors. Such an assessment is more appropriate for health system leaders and policymakers. Further policy around managing one’s CO, institutional guidance and education should be offered to conscientious objectors. This should explicitly cover how health professionals can effectively preserve and protect their moral integrity, whilst ensuring their CO does not impede patients’ access. Further empirical research that explores CO and VAD and its regulatory consequences should be undertaken. Such research is particularly salient currently as more Australian jurisdictions seek to legalize and implement VAD.

## Data Availability

Participant quotes supporting this article’s specific findings have been included in this article in their raw de-identified form where relevant. The broader dataset of the study is not publicly available and not available on request due to the sensitive nature of the data collected and due to ethics application restrictions.

## Abbreviations

CO: Conscientious objection
CMH: Casey Michelle Haining
LAK: Louise Anne Keogh
VAD: Voluntary assisted dying
